# The Novel Actions of the Metabolite GnRH-(1-5) are Mediated by a G Protein-Coupled Receptor

**DOI:** 10.3389/fendo.2013.00083

**Published:** 2013-07-08

**Authors:** Darwin Omar Larco, Nina Nashat Semsarzadeh, Madelaine Cho-Clark, Shaila K. Mani, T. John Wu

**Affiliations:** ^1^Program in Molecular and Cellular Biology, Uniformed Services University of the Health Sciences, Bethesda, MD, USA; ^2^Department of Obstetrics and Gynecology, Uniformed Services University of the Health Sciences, Bethesda, MD, USA; ^3^Department of Molecular and Cellular Biology, Baylor College of Medicine, Houston, TX, USA; ^4^Department of Neuroscience, Baylor College of Medicine, Houston, TX, USA

**Keywords:** GPR173, GnRH, GPCR, migration, EP24.15, SREB3

## Abstract

The gonadotropin-releasing hormone (GnRH) was originally isolated from the mammalian hypothalamus for its role as the primary regulator of reproductive function. Since its discovery, GnRH has also been shown to be located in non-hypothalamic tissues and is known to have diverse functions. Although the regulation of GnRH synthesis and release has been extensively studied, there is additional evidence to suggest that the processing of GnRH to the metabolite GnRH-(1-5) represents another layer of regulation. The focus of this review will be on the current evidence for the action of the pentapeptide metabolite GnRH-(1-5) in regulating cellular migration. We discuss the potential role of GnRH-(1-5) in regulating GnRH neuronal migration during development. Furthermore, we demonstrate these actions are mediated by the activation of a G protein-coupled receptor. Our findings suggest that GnRH-(1-5) may play a developmental function in addition to regulating developing cells.

## Introduction

The regulation of reproductive function is dependent on the proper coordination between the three axes of reproduction: the hypothalamus, anterior pituitary, and the gonads [hypothalamic-pituitary-gonadal (HPG) axis]. Gonadotropin-releasing hormone (GnRH) is a key regulator of this axis and thus of reproductive function and behavior. GnRH has 23 known isoforms, which are present in both mammalian and non-mammalian species (1). The N- and C-terminal sequences of GnRH, pGlu-His-Trp-Ser, and Pro-Gly NH_2_ respectively, have been highly conserved throughout evolution reinforcing the essential role GnRH plays in reproduction (1).

Neurons that synthesize GnRH are widely distributed in the basal forebrain and project to the median eminence where GnRH is released in a pulsatile manner into the hypophyseal portal vessels. Secreted GnRH stimulates the synthesis and secretion of the gonadotropins, luteinizing hormone (LH), and follicle-stimulating hormone (FSH), in the anterior pituitary. These gonadotropins subsequently exert their effects at the gonadal level to regulate the secretion of steroid hormones. In the female, LH and FSH act on the ovary to facilitate follicular maturation and to regulate the secretion of estrogen and progesterone, which act as feedback modulators of the hypothalamic-pituitary axis. Estrogen has been shown to both stimulate and inhibit GnRH release depending on the stage of the estrus cycle (2–4). Furthermore, GnRH can regulate its own synthesis and secretion via an autocrine mechanism, which can be influenced by other neuropeptides (5, 6). Despite significant research clarifying the HPG axis, the integration of signals required for normal function of the GnRH neuroendocrine axis is not completely understood.

In our previous work, we have shown that the metabolite of GnRH, GnRH-(1-5), is biologically active and may serve as another regulatory factor of the HPG axis. It has been established that GnRH neurons originate outside of the central nervous system (CNS) and migrate to its final position in the basal forebrain (7, 8). Along this migratory pathway, GnRH is cleaved by the zinc metalloendopeptidase EC 3.4.24.15 (abbreviated EP24.15), to generate GnRH-(1-5) (9–11). Although neuropeptidases have been thought to degrade proteins to cease further peptide action, GnRH-(1-5) has been shown to facilitate reproductive behavior (12) and to increase GnRH mRNA levels (13). More recently, GnRH-(1-5) was demonstrated to inhibit the migration of GN11 cells, a GnRH secreting cell line, by binding the G protein-coupled receptor (GPCR), GPR173 (14). These biologically active roles of GnRH-(1-5) may implicate the peptide as a regulator of the HPG axis rather than simply a metabolic byproduct.

## Development of the GnRH Neuroendocrine System

**Figure 1:**
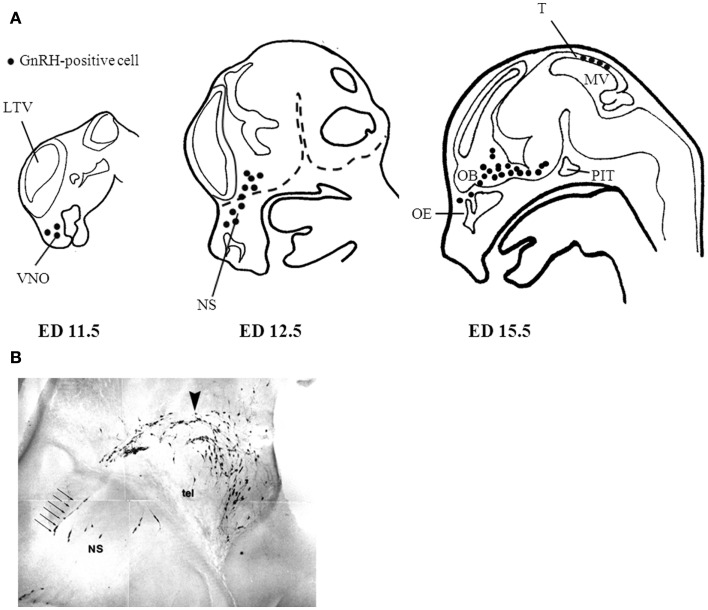
**Gonadotropin-releasing hormone neuronal migratory route during development**. **(A)** GnRH neurons are born in the nasal placode near the vomeronasal organ (VMO) at approximately embryonic day (ED) 11.5 in the mouse. As development progresses GnRH neurons begin to enter the CNS (dashed lines) at ED12.5 and begin to descend within the basal forebrain region at ED15.5. **(B)** Most GnRH neurons are within the CNS at ED15.5 however the remaining GnRH neurons in the nasal septum (NS) have a morphologically distinct migratory pattern relative to neurons found near the telencephalon (tel). Arrow indicates descending GnRH neurons of a mouse ED15.5 brain (Wu, TJ unpublished data). LTV, left telencephalic vesicle; OB, olfactory bulb; OE, olfactory epithelium; PIT, pituitary; MV, mesencephalic vesicle; T, tectum.

Initial studies by Schwanzel-Fukuda demonstrated that GnRH neurons originate outside the CNS in the nasal region (15). Subsequent studies validated these observations showing that GnRH neurons migrate from the nasal placode traversing the cribriform plate to target the basal forebrain (7, 8). In the developing mouse, GnRH immunoreactivity is detected as early as embryonic day (ED) 10.5 in the vomeronasal region (15). By ED 15, most GnRH neurons have already migrated along vomeronasal tract to the prepoptic area (7, 15). A subpopulation of GnRH positive cells are localized in the tectum near the mesencephalic vesicle peaking at ED 15; however, they cease to exist in the adult and their function has not been elucidated (16). The primary GnRH neuronal population in adulthood is estimated to be near 800 cells distributed throughout the basal forebrain (7, 8, 17). Figure 1A shows the GnRH neuronal migratory route during different developmental stages in the mouse. At approximately ED 15.5, most GnRH neurons have crossed the cribriform plate into the basal forebrain. Interestingly, GnRH positive cells near the nasal region migrate radially, which is in contrast to their tangential migratory pattern once these neurons are in the CNS (Figure 1B). The proper migration of GnRH neurons during development requires the integration of multiple guidance cues in part mediated through the activation of certain GPCRs including prokineticin receptor-2 (PROKR2) (18), and CXCR4 (19). Additionally, the migratory rate of GnRH neurons seems to be another layer of regulation as there is a brief delay in migration as they begin to traverse the cribriform plate to enter the CNS. This delay may serve to aid in the transition between the nasal region and the CNS to properly respond to a changing microenvironment and migratory trajectory (20). A more comprehensive overview of the development of the GnRH neuroendocrine system can be found in the reviews in (21) and (22).

## Hypogonadotropic Hypogonadism

Defects in the HPG axis lead to the development of various types of hypogonadotropic hypogonadism (HH) where the onset of puberty and fertility are impaired. There are a number of genes implicated in the pathogenesis of HH, including mutations in the genes encoding the GnRH peptide and its receptor. Mutations associated with these genes are generally classified as normosmic HH (nHH) where GnRH signaling is impaired yet olfactory senses remain intact (23). Other forms of HH exist where patients suffer from reduced olfaction in addition to deficiencies in pubertal development and fertility. This form of HH also called Kallmann Syndrome (KS) is attributed to a defect in GnRH neuronal migration. Specifically, precursor olfaction and GnRH neurons emanating from the olfactory placode remain arrested in the cribriform plate during development, never entering the CNS to reach their target sites (24). Patients diagnosed with nHH or KS have reduced levels of circulating gonadotrophins, LH, and FSH, which would normally stimulate the secretion of steroid hormones from the testes or ovaries. In turn, these patients suffer from delayed pubertal onset and reduced gonadal function among other pathologies (23, 25–28).

Recent studies focusing on the molecular basis for KS implicate genes believed to aid in both the origination and migration of GnRH neurons during development such as FGFR1, FGF8, and KAL1 (29). Disruption of FGFR1 signaling was demonstrated to lead to the development of KS (30). FGFR1 and its ligand are required for the proper birth and maintenance of GnRH neurons during embryogenesis; knockdown of both genes results in the absence of GnRH neurons (31). In addition to the complete absence of GnRH neurons, disruption of neuronal migration has also been identified as a cause of KS. Observation of a Kallmann’s fetal brain revealed GnRH and olfactory neurons resided in the nasal cavity and failed to cross the cribriform plate to enter the CNS (32). This improper migration of GnRH neurons was attributed to the deletion of the KAL1 gene located on the X chromosome at Xp22.3. More recently, *in vitro* studies indicate the product of KAL1 acts as a chemoattractant for immortalized premigratory GnRH neurons (33) by associating with cell surface heparin sulfate proteoglycans (34). Interestingly, many signaling cues required for normal GnRH neuronal migration require the activation of GPCRs including PROKR2, which has been linked to the development of KS (35). Mutations localized in the intracellular loop 1 of PROKR2 have been identified in patients with KS and is attributed to aberrant receptor membrane localization and receptor activity (36). However, complete identification of all factors contributing to the proper migration of GnRH neurons is still limited since the majority of KS cases have yet to be linked to a genetic defect (37).

Patients with nHH harboring mutations in genes encoding GnRH or its receptor GnRHR do not have obvious anatomical aberrations of the hypothalamus that would suggest a defect in neuronal migration; yet they continue to suffer from delayed pubertal development. Recently, a frameshift mutation in the GnRH gene was identified in teenage siblings who had intact olfaction but suffered from underdeveloped gonadal function. GnRH treatment in one of the siblings increased circulating LH levels (38, 39), indicating that the GnRHR was responsive to exogenous GnRH administration. These findings are recapitulated in studies using the hypogonadal (HPG) mouse where the GnRH gene is no longer expressed; however still retain the ability to respond to exogenous GnRH (40, 41). Furthermore, HPG mice receiving preoptic transplantations containing GnRH neurons induced episodic LH secretion; thus rescuing the knockout phenotype and indicating that the developmental expression of GnRH is not required for a functional GnRH pulse generator (42).

Apart from defective GnRH gene expression, a significant number of other patients with nHH have been shown to contain various types of mutations in the gene encoding its receptor the GnRHR leading to complete or partial loss of receptor activity (23, 43, 44). Like the HPG mouse, GnRHR deletion in mouse models disrupts pubertal onset and gonadal function; yet, exogenous GnRH treatment does not rescue the phenotype and demonstrates that GnRH function requires proper GnRHR signaling (45). Furthermore, these studies paralleled observations seen in patients with nHH linked to defective GnRHR activity where the distribution of GnRH neurons in the brain was normal (45). In terms of receptor activity, the severity of hypogonadism in patients depends on the specific mutation within the GnRHR gene (46, 47). In certain cases low levels of pulsatile LH secretion are detected whereas others are completely unresponsive to exogenous GnRH administration and thus gonadotropin levels are absent (43). Future studies will need to address the functional relevance of the specific GnRHR mutations to potentially develop therapeutics that may overcome or ameliorate the reproductive deficits observed in these patients.

## GnRH Metabolism

**Figure 2:**
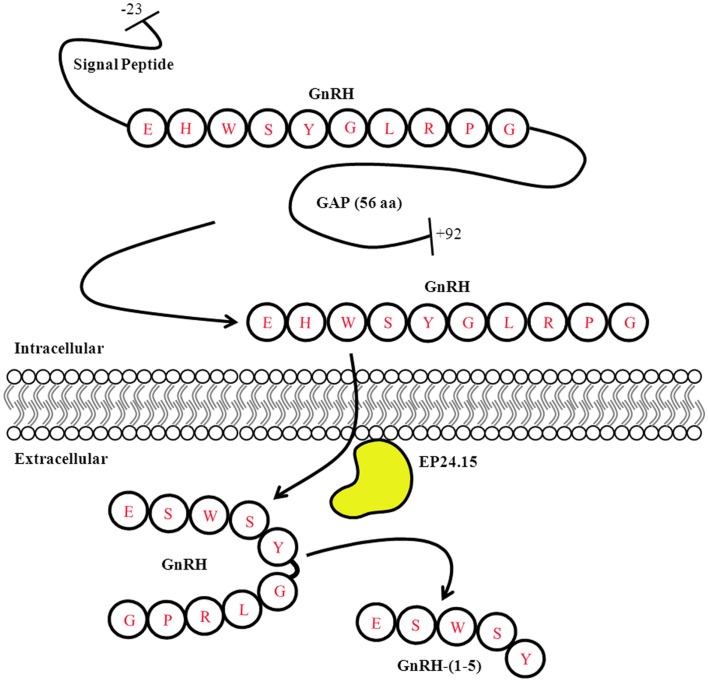
**Gonadotropin-releasing hormone Peptide Processing**. The prepro-GnRH is processed intracellularly to generate the mature GnRH peptide. In the extracellular matrix, GnRH is metabolized in a two step mechanism generating the metabolite GnRH-(1-5). Modified from Roberts et al. (11).

A number of neuropeptides can undergo enzyme-mediated processing – whether intracellularly or extracellularly – leading to different functional roles. For example, the precursor polypeptide proopiomelanocortin (POMC) can undergo several intracellular post-translation modifications (PTM) that give rise to different mature peptides such as ACTH, α-MSH, and β-endorphin (48). Although these peptides are synthesized from the same parent peptide they do not necessarily mediate the same functions. Furthermore, their processing is dependent on the tissue where they are expressed (49). Similarly, the prepro-GnRH undergoes several intracellular modifications before giving rise to the mature form of the GnRH peptide. The prepro-GnRH is 92 amino acids with an N-terminal signal sequence that is removed to generate the pro-GnRH peptide. The decapeptide GnRH is located closer to the N-terminus while the C-terminal region contains the GnRH-associated peptide (GAP). Both fragments are separated by a cleavage site that allows for further processing to generate the mature forms of the GnRH and GAP peptides (50) (Figure 2). The function of GAP is not clear but it has been shown to regulate prolactin, LH, and FSH secretion (51). Furthermore, it is possible GAP is metabolized into smaller peptide fragments that have biological activity; however this remains to be determined.

The previous notion that neuropeptidases in the extracellular milieu degrade peptides to cease further biological activity has been modified due to the growing evidence that suggests otherwise (11, 52, 53). Metabolized peptide products have been shown to regulate a variety of functions independent of the parent peptide. For example, the C-terminal fragments degraded from the full-length arginine vasopressin have no effect on water retention but modulate learning behavior and memory formation (54, 55). Furthermore, there is evidence suggesting that metabolized fragments can regulate a more specific function from that of the parent peptide. This is the case for bradykinin-(1-5), which only inhibits thrombin-induced platelet aggregation, but does not induce vasodilation like its parent peptide bradykinin (56). Collectively, these findings highlight another level of complexity where peptidases can be viewed as activating enzymes that participate in the regulation of a wide variety of processes in the CNS.

In the extracellular matrix, GnRH is metabolized by the zinc metalloendopeptidase, EC 3.4.24.25 (EP24.15) (Figure 2). EP24.15, also commonly referred to as thimet oligopeptidase, is a hydrolase (3), which acts on peptide bonds (4) requiring Zn^2+^ as a coactivator (24) and thiol-mediated activation (15). EP24.15 is most prominently expressed in the brain, testis, and pituitary gland. Enzymes in this family contain the HEXXH motif in the active site common in zinc metallopeptidases and are known to hydrolyze peptide bonds in substrates less than 40 amino acids in length (52). EP24.15 has specificity for substrates less than 17 amino acids long and prefers substrates with hydrophobic residues at the P1, P2, and P3′ positions (52, 57). Regulation of EP24.15 enzymatic action is regulated by various mechanisms including thiol activation (58) and phosphorylation (59). Although some members of the family are membrane-bound, EP24.15 lacks a transmembrane domain and is primarily found in a soluble form in both the brain and periphery. EP24.15 has also been observed to have an extracellular association with the plasma membrane where it displays ectoenzymatic activity (60). Despite this finding, analysis of the EP24.15 cDNA sequence does not contain a motif indicative of a plasma membrane association (60). Further examination of EP24.15 associating with the extracellular face of the plasma membrane revealed that it localizes with lipid rafts (61). This localization with lipid rafts could produce a microenvironment in which EP24.15 can readily access its substrates, including GnRH, and alter signaling pathways (61).

To ensure the proper hydrolysis of GnRH to produce GnRH-(1-5), EP24.15 requires both zinc in its active site and protein kinase A (PKA). PKA phosphorylates EP24.15 at serine residue 644, which actually decreases the enzyme’s affinity for GnRH. Despite its reduced affinity, when EP24.15 does bind GnRH, it produces substrate very rapidly (59). This mechanism suggests that the enzyme can handle large concentrations of peptide without becoming saturated, which is important due to the pulsatile nature of GnRH release (53, 59). In addition to the activation of the enzyme, GnRH must also assume a specific conformation to properly dock at the active site of EP24.15. The N- and C-terminus of the pentapeptide come together to assume a βII’-type turn conformation. It has been shown that the sixth amino acid residue of GnRH, a highly conserved glycine, is essential for this folding. When the sixth residue is replaced by with d-amino acids, as in GnRH analogs like leuprolide, the peptide is no longer hydrolyzed by EP24.15 and GnRH-(1-5) is not produced. The full-length GnRH is metabolized in a two-step mechanism. First, the glycine residue at the 10th position is removed by a prolyl endopeptidase to generate GnRH-(1-9). Subsequently, EP24.15 cleaves GnRH-(1-9) at the 5th and 6th position thereby forming the metabolite GnRH-(1-5) (52, 58, 62) (Figure 2).

Apart from GnRH processing, EP24.15 is also involved in the metabolism of other peptides including bradykinin, neurotensin, and angiotensin I, which may produce different biological outcomes (53, 63). For example, EP24.15 cleaves angiotensin I to angiotensin-(1-7), which has the opposite effect on blood pressure relative to his parent peptide (63). This finding suggests that EP24.15 can process various peptides that can oppose the actions of the parent peptide. Similarly, we have shown that GnRH-(1-5) stimulates GnRH mRNA expression in GnRH neurons while treatment with the full-length GnRH inhibits its expression (13). Therefore in mammals EP24.15 may have a role in regulating reproductive behavior by processing GnRH to GnRH-(1-5). In a previous study, we have shown immunoneutralization of EP24.15 in animals treated centrally with GnRH reversed the ability of GnRH to facilitate lordosis behavior in the female rat. Furthermore, female rats treated with GnRH-(1-5) facilitated the lordosis response (12), suggesting that this behavior can indirectly be regulated by EP24.15 via of its processing of GnRH to form the bioactive GnRH-(1-5). This is further reinforced by evidence indicating that exposure to the steroid hormone estrogen regulates EP24.15 levels in brain regions implicated in reproductive function such as the ventromedial nucleus of the hypothalamus (64) and in the lateral external layer of the median eminence where GnRH neurons terminate (8). Collectively, these studies suggest that EP24.15 is an important processor of GnRH to form the bioactive GnRH-(1-5).

## GnRH-(1-5)-Mediated Activation of GPR173

The endopeptidase EP24.15 is expressed early in development along the GnRH migratory path where the levels of GnRH-(1-5) have been detected (9). Thus the availability of GnRH-(1-5) and its localization with immature GnRH neurons suggest that it may play a regulatory role in the migration of GnRH neurons prior to entering the CNS through the cribriform plate. We have previously demonstrated that GnRH-(1-5), generated by EP24.15 activity, has functional roles by stimulating GnRH mRNA expression in immortalized GnRH neurons (13); and facilitating lordosis behavior in rodents (12). However, the role of GnRH-(1-5) during development specifically on GnRH neurons has not been established. Recently, we implicated GnRH-(1-5) as regulator of GnRH neuronal migration using the immortalized GnRH secreting cell line, the GN11 cell, as an *in vitro* model (14). We demonstrated that GnRH-(1-5) inhibited GN11 cellular migration via the activation of GPR173 (14). Physiologically this finding may in part contribute to the brief delay observed in the migration rate of GnRH neurons as they begin to transition into the CNS (20). In addition to our findings, it is plausible that a physical barrier such as the cribriform plate impedes the migration of the GnRH neurons. Also, changes in the ECM or surrounding cells could be another justification for the decrease in migration. For example, it has been demonstrated that GABAergic signaling from surrounding neurons inhibits GnRH neuronal movement in the nasal compartment (65). Regardless, our studies implicate an endogenous ligand for the orphan receptor GPR173 indicating that the delay in migratory rate may indeed serve an important function to potentially aid in GnRH neuronal maturation or to facilitate the proper coordination of these neurons to their targets within the CNS (20).

Our previous work and unpublished observations suggest that GPR173 levels are developmentally regulated specifically in sites of GnRH-(1-5) action including the nasal region of embryonic mice (14) and in the hypothalamus. Additionally, these findings are in accord with high-throughput expression and *in situ* analyses demonstrating the presence of GPR173 is enriched in these regions relative to other sites in the brain and in the periphery (66, 67). GPR173 belongs to a small subfamily of GPCRs known as the Super Conserved Receptor Expressed in Brain (SREB) family. Only two other SREB proteins have been identified: GPR27 and GPR85 (68). The SREB proteins are expressed primarily in the brain and genital organs (68). Functional studies reveal that GPR27 regulates the production of insulin (69, 70) while GPR85 is thought to play a role in neurogenesis (71). Although no known ligands have been found to activate the SREB receptors with GPR173 as the exception, they are thought to be aminergic receptors due to their sequence similarity to other such GPCRs (68). This is an interesting concept since GnRH-(1-5) is a pentapeptide with an imidazole functional group provided by the second N-terminal amino acid histidine, which may play a role in the activation of GPR173.

**Figure 3:**
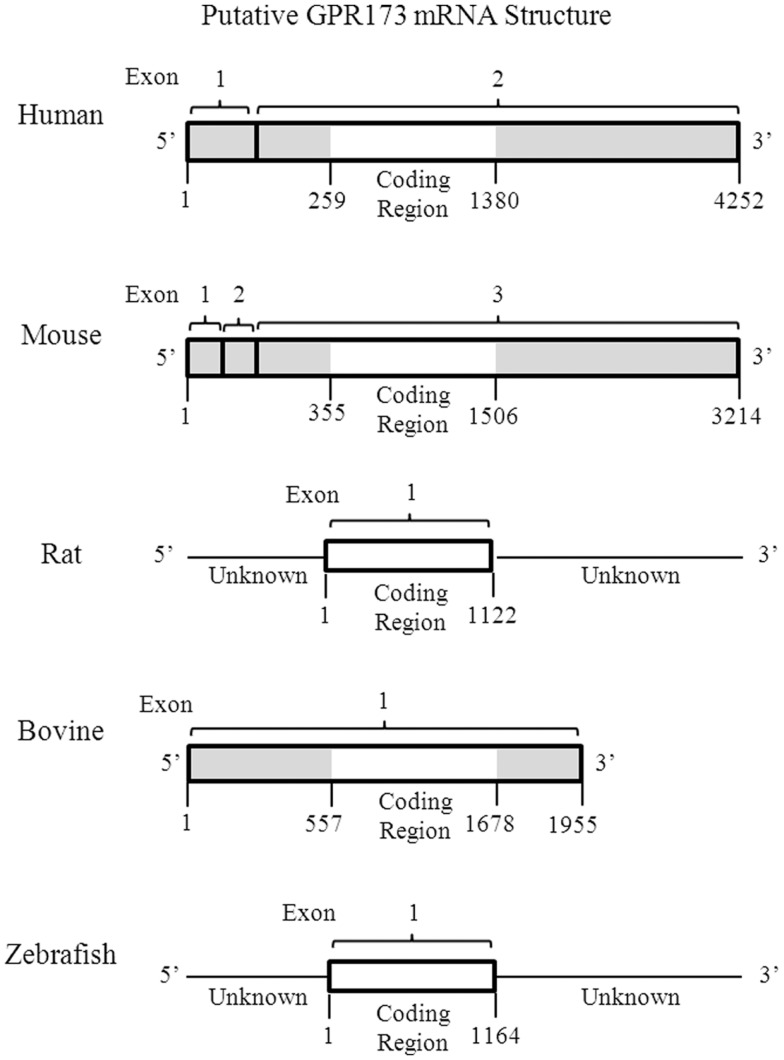
**Putative mRNA Structure of GPR173 across Species**. Analysis of the putative GPR173 mRNA structure across species reveals a significant degree of conservation within the coding region. These structures were generated from NCBI website (Bethesda, MD, USA). The mRNA accession number can be found inTable 1.

**Figure 4:**
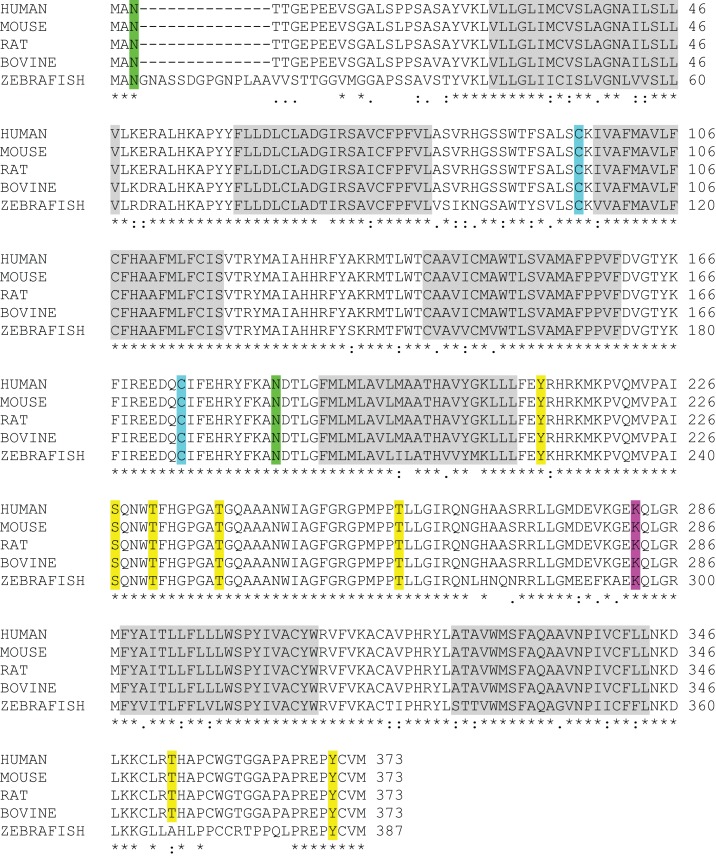
**GPR173 Protein Sequence Alignment**. A protein sequence analysis of GPR173 reveals a high degree of conservation across species. Additionally, many sites within the intracellular loop 3 are putative phosphorylation sites (yellow) with a conserved lysine residue (purple), which may undergo ubiquitination. A highly conserved arginine residue (green) in the N-terminal tail indicates a putative site for N-glycosylation. “*” indicate conserved residues; “.” or “:” indicate similarity between residues. Shaded regions indicate putative transmembrane spanning domains (TM). Sequence alignment analysis was conducted by using the UniProt consortium (www.uniprot.org).

One of the most intriguing aspects of GPR173 is its conservation among vertebrate species. Orthologs of GPR173 have been identified in rats, mice, bovine, and the zebrafish species (Figure 3). At the mRNA level, the protein-coding regions of many species share a significant degree of homology relative to the human GPR173 (Table 1). Similarly, analysis of the human, mouse, rat, and zebrafish orthologs of GPR173 show a high degree of conservation and reveal similar putative sites of PTM (Figure 4). Like other highly conserved proteins, this degree of conservation throughout evolution suggests an imperative physiological purpose. GnRH is a neuropeptide that has been conserved throughout evolution in both vertebrates and invertebrates and is known to be essential to reproduction in vertebrates – a function, without which, there would be no propagation (72). The conservation of GPR173 throughout the evolution of vertebrates suggests its function to be important, if not indispensible.

**Table 1:** **Percent homology of the GPR173 mRNA sequence across species**.

	Mouse NM_027543.3	Rat NM_022255.1	Bovine NM_001015604.1	Zebrafish NM_131498.1
Human NM_018969.5 (%)	95	95	95	77

*The percent homology was calculated using the coding region of the putative GPR173 mRNA sequence of each species relative to the human sequence. Sequence information and the calculation of homology were obtained from the NCBI website (http://www.ncbi.nlm.nih.gov/pubmed) and the NCBI Blast website (http://blast.ncbi.nlm.nih.gov/Blast.cgi), respectively. The NCBI accession ID is indicated below each species*.

**Figure 5:**
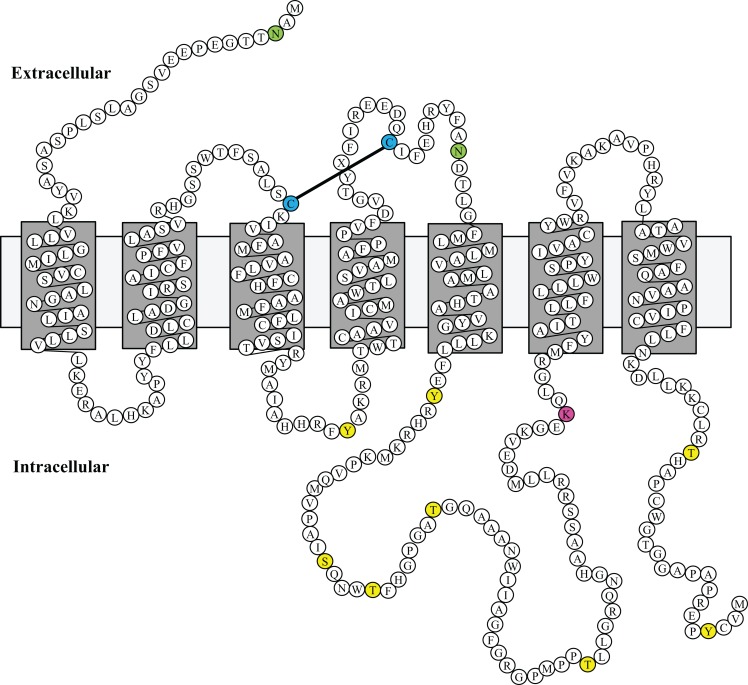
**Putative Model of Mouse GPR173 Disposition in the Membrane**. The structure sketch map of Mouse GPR173 shows a large intracellular loop with multiple sites that may undergo post-translational modification. Yellow denotes putative phosphorylation site; purple denotes putative ubiquitination site; and green denotes putative N-glycosylation site. This model of GPR173 was generated with the aid of the UniProt consortium (www.uniprot.org).

Using a bioinformatic approach, we examined the GPR173 sequence for likely PTM sites that regulate receptor activity and function. There are seven putative phosphorylation sites (Figure 4), many of which are in the large third cytoplasmic loop (Figure 5). This is of significance since phosphorylation typically negatively regulates GPCRs by desensitization and endocytosis, best exemplified by the β-adrenergic receptor (βAR). Upon epinephrine binding to the βAR, a cascade of events occur that lead to sequential activation of PKA, β-adrenergic receptor kinase (βARK), and the receptor internalization and desensitization (73–75). The mechanism of internalization typically involves the recruitment of adaptor proteins such as β-arrestin initially believed to solely mediate GPCR internalization. However, recent evidence suggests β-arrestin may play a more diverse function in regulating GPCR function. For example, the activation of the parathyroid hormone receptor 1 (PHR1) leads to the formation of a complex consisting of PHR1, β-arrestin, and Gβγ subunits to enhance cAMP formation rather than cease receptor activity (76). Additionally, βAR has also been shown to deviate from the GPCR canonical signaling pathway to recruit β-arrestin and c-Src subsequently leading to the activation of the ERK pathway (77). Whether any of the potential sites within the intracellular loops of GPR173 are indeed phosphorylated remains to be determined; however, the high degree of conservation of these motifs across species indicates they may play a role in receptor function (Figure 4). Mutagenesis studies will be instrumental in examining the role played by each of these motifs and how they regulate GPR173 activity in response to GnRH-(1-5) during development and in adult function.

Another potential site for PTM within the accessible areas of GPR173 is an asparagine residue in the extracellular N-terminal tail (Figure 5). This site is a candidate for N-glycosylation, which may facilitate receptor interaction with the extracellular milieu or assist in ligand recruitment. Previous studies have identified two glycosylation sites (Asn4 and Asn18) within the GnRHR to be critical for receptor expression and stability in the plasma membrane (78). Additionally, N-glycosylation sites on the Luteinizing Hormone Receptor (LHR) at Asn173 and Asn152 were demonstrated to be essential in the binding of LH to its receptor (79). Lastly, there is a lysine in the third cytoplasmic loop that could potentially be a target of ubiquitination (Figure 5). Ubiquitination has long been associated with the down regulation of GPCR activity via receptor endocytosis and degradation, but recent studies suggest that ubiquitination plays a role in the trafficking and positive regulation of certain GPCRs (80). In some instances, ubiquitination-mediated internalization of GPCRs facilitates the formation of complexes important in propagating a signal rather than ceasing activity (80). This is best exemplified by the co-internalization of the angiotensin (AT1A) receptor and activated pERK-1/2 into an endosome to allow for sustained activity of pERK-1/2 (80). Whether this type of regulation or any of the PTMs mentioned are related to GPR173 function and activity remains to be addressed.

Our previous work implicates the GnRH metabolite GnRH-(1-5) in the regulation of GnRH neuronal migration. The GnRH-(1-5) mechanism is complex involving the interaction of other growth factors, which serve to stimulate migration. This cooperation between stimulators of migration and the inhibitory action of GnRH-(1-5) is likely important at the region separating the nasal compartment from the CNS where there is a brief delay in GnRH neuronal migration. GnRH-(1-5) binding to GPR173 may serve to modulate the migratory rate of GnRH neurons to potentially prime them for entry into the CNS or to facilitate the maturation of these neurons. Our understanding of the mechanism responsible for the integration of all signaling inputs to properly establish the GnRH neuronal population in the basal forebrain remains to be completely elucidated. Our studies on GnRH-(1-5) indicate GPR173 is another factor that may contribute to the development of the HPG axis. Future studies will need to verify the physiological significance of GnRH-(1-5) activating GPR173 during embryogenesis in addition to its role in the adult since our initial studies indicate GnRH-(1-5) plays other roles in reproductive biology.

## Conflict of Interest Statement

The authors declare that the research was conducted in the absence of any commercial or financial relationships that could be construed as a potential conflict of interest.
